# Supplementary material for: Prevalence of obesity according to body mass index, waist circumference, and waist-to-height ratio in Peru: A systematic review and meta-analysis
Source: Obes Pillars. 2025 Feb 8;13:100166. doi: 10.1016/j.obpill.2025.100166 (PMC11869839; doi:10.1016/j.obpill.2025.100166)
Supplement: Multimedia component 1 [file mmc1.docx]

Supplementary file 1

| Search strategy in PUBMED | |
| --- | --- |
| #1 | "Obesity"[Mesh] OR "Obesity"[tiab] OR "Body Mass Index"[Mesh] OR "Body Mass Index"[tiab] OR "BMI" OR "Overweight"[Mesh] OR "Overweight"[tiab] OR "Adiposity"[Mesh] OR "Adiposity"[tiab] OR "Waist-Height Ratio" OR "Waist Circumference" |
| #2 | "Peru" |
| #3 | #1 AND #2 AND #3 |
| Search strategy in EMBASE | |
| #1 | ('obesity'/exp OR 'adipose tissue hyperplasia' OR 'adipositas' OR 'adiposity' OR 'alimentary obesity' OR 'body weight, excess' OR 'corpulency' OR 'fat overload syndrome' OR 'nutritional obesity' OR 'obesitas' OR 'obesity' OR 'overweight' OR 'waist circumference'/exp OR 'waist circumference' OR 'waist size' OR 'waist to height ratio'/exp OR 'waist height ratio' OR 'waist to height ratio' OR 'waist-height ratio') |
| #2 | ('peru'/exp OR 'peru' OR 'peruvian'/exp OR 'peruvian' OR 'peruvians') |
| #3 | #1 AND #2 AND #3 |
| Search strategy in Scopus | |
| #1 | ( TITLE-ABS-KEY (( "Obesity" OR "Body Mass Index" OR "BMI" OR "Overweight" OR "Adiposity" OR " Waist-Hip Ratio" OR " Waist Circumference" )) |
| #2 | TITLE-ABS-KEY (( "Peru" OR "peruvian" OR peruvians ))) |
| #3 | #1 AND #2 AND #3 |
| Search strategy in Web of Science | |
| #1 | (TS=(("Obesity" OR "Body Mass Index" OR "BMI" OR "Overweight" OR "Adiposity" OR "Waist-Height Ratio" OR " Waist Circumference"))) |
| #2 | TS=(("Peru" OR "peruvian" OR peruvians)) |
| #3 | #1 AND #2 AND #3 |
| Search strategy in LILACS/Scielo | |
| #1 | ((obesidad) OR (obeso) OR (sobrepeso) OR (adiposidad) OR (IMC) OR ("índice de masa corporal" OR "Circunferencia de la Cintura" OR "perímetro abdominal" OR "perímetro de cintura" OR "circunderencia abdominal" OR "Relación Cintura-Estatura")) |
| #2 | ((Perú) OR (Peru) OR (peruanos)) |
| #3 | #1 AND #2 AND #3 |
